# Draft Genome Sequences of Four *Enterococcus faecium* Strains Isolated from Argentine Cheese

**DOI:** 10.1128/genomeA.01576-15

**Published:** 2016-02-04

**Authors:** Gabriela P. Martino, Ingrid M. Quintana, Martín Espariz, Victor S. Blancato, Gabriel Gallina Nizo, Luis Esteban, Christian Magni

**Affiliations:** a Laboratorio de Fisiología y Genética de Bacterias Lácticas, Instituto de Biología Molecular y Celular de Rosario (IBR-CONICET), Rosario, Argentina; b Laboratorio Biotecnología e Inocuidad de los Alimentos, Facultad de Ciencias Bioquímicas y Farmacéuticas, Universidad Nacional de Rosario (UNR), Rosario, Argentina; c Facultad de Ciencias Médicas, UNR, Rosario, Argentina

## Abstract

We report the draft genome sequences of four *Enterococcus faecium* strains isolated from Argentine regional cheeses. These strains were selected based on their technological properties, i.e., their ability to produce aroma compounds (diacetyl, acetoin, and 2,3-butanediol) from citrate. The goal of our study is to provide further genetic evidence for the rational selection of enterococci strains based on their pheno- and genotype in order to be used in cheese production.

## GENOME ANNOUNCEMENT

*Enterococcus faecium* is part of the human and animal gut microbiota. Also, it is widely distributed in diverse habitats such as soil, water, vegetables, and food. These enterococci are an important source of biodiversity in traditional fermented foods (1, 2). However, they have emerged in recent years as opportunistic nosocomial pathogens (3, 4). The genus *Enterococcus* comprises Gram-positive cocci that are catalase-negative, able to grow at 6.5% wt/vol NaCl, 45°C, pH 9.6, in bile esculin (40%) agar medium, tolerant to tellurite, and positive for leucine aminopeptidase and pyrrolidonyl arylamidase enzymes. Here, we report four strains isolated from regional cheeses pheno- and genotypically characterized as *E. faecium* (5). To gain better insight into the genetic diversity of the four strains, their whole-genome sequences were determined. In Table 1 we summarize the whole-genome data of the four enterococci strains: IQ110, IQ23, GM70, and GM75. Citrate metabolism in these strains was described by Martino et al. (5). Briefly, genetic evidence of citrate metabolism was obtained through PCR analysis determining the presence of the citrate lyase complex (*citE* and *citF* genes). Also, citrate radioactive uptake was determined in resting cells of *E. faecium* strains, as well as C4 compound production in medium supplemented with citrate. Cellular aggregates were detected in liquid medium and remained insoluble despite mechanical disruption (5).

**TABLE 1:** Summary of information for the whole genomes of four *Enterococcus faecium* strains

Strain	Phenotype/genotype^a^	Genome size (bp)^b^	GC (%)^b^	CDSs/RNAs^b^	Accession no.
IQ23	Cit^+^, Agg^+^	3,124,007	37.7	3,052/69	LKPF00000000
IQ110	Cit^−^, Agg^−^	2,757,341	37.9	2,776/69	LKPG00000000
GM70	Cit^+^, Agg^−^	2,696,915	38.0	2,741/65	LKPH00000000
GM75	Cit^+^ Agg^−^	2,848,961	38.1	2,986/92	LKPI00000000

^a^ Cit^+^: citrate metabolism was determined by PCR amplification of the citrate lyase genes *citE* and *citF*, citrate uptake, and Voges-Proskaur reaction (5). Agg^+^ phenotype: cellular aggregates insoluble despite mechanical disruption.

^b^ The genome size, GC content, and putative coding sequences (CDSs)/RNAs were predicted by RAST automated service (8).

Genomic DNA of the *E. faecium* strains was extracted using the Wizard genomic DNA purification kit (Promega). The genome sequences were determined using an Illumina HiSeq 2000 platform (MR DNA). *De novo* assembly was performed with SeqMan NGen (DNASTAR Inc.). Through BLASTn analysis (all versus all), contigs shorter than 1,000 bp, with higher than 99% identity to other sequences and already contained in a longer contig were deleted. The remaining contigs were ordered and oriented with Advanced Pipmaker (6) and Mauve version 2.3.1 (7). Genome annotation was performed using RAST (Rapid Annotations using Subsystem Technology) (8).

This report will contribute to understanding the positive and negative impact of *E. faecium* in cheese fermentation. Furthermore, comparative whole-genomic analysis of enterococci will improve our knowledge of this controversial group of microorganisms and the evolutionary mechanisms involved in their adaptation to specific niches, and it will assist in evaluating the putative use of selected *E. faecium* strains as adjunct cultures for cheese production.

### Nucleotide sequence accession numbers.

The draft genome sequences of the *Enterococcus faecium* strains described here have been deposited at DDBJ/EMBL/GenBank under the accession numbers given in Table 1.
